# Anti-cancer effect of palmitic acid against the endometrial cancer progression via inducing ferroptosis

**DOI:** 10.1016/j.bbrep.2026.102499

**Published:** 2026-02-10

**Authors:** Shuting Lan, Xiaomei Sun, Qiyuan Bo, Suriguga Wang, Yifan Qin, Jianying Mao

**Affiliations:** a Baotou Medical College, Inner Mongolia University of Science and Technology, Baotou, Inner Mongolia, 014040, China; b Department of Internal Medicine, Gu Yang People's Hospital, Baotou, Inner Mongolia, 014200, China; c Department of Gynecology and Obstetrics, Mongolian Hospital of Xilingol, Xilinhot, Inner Mongolia, 026000, China; d Department of Gynecology and Obstetrics, Inner Mongolia People's Hospital, Hohhot, Inner Mongolia, 010017, China

**Keywords:** Palmitic acid, Endometrial cancer, Ferroptosis, Metabolic reprogramming, Tumor progression

## Abstract

**Background:**

The critical role of metabolic reprogramming as a potential therapeutic target in the management of endometrial cancer (EC) progression requires further investigation. This study investigated the anti-cancer effect of palmitic acid (PA) on EC progression using cellular and xenograft models, combined with integrated transcriptomic and metabolomic analyses to elucidate the molecular pathways through which PA induces ferroptosis and inhibits tumor growth.

**Methods:**

The anti-cancer effects of PA were assessed through comprehensive *in vitro* assays, including cell viability, proliferation, migration, invasion, adhesion, clonogenicity, cell-cycle distribution, apoptosis, and epithelial-mesenchymal transition (EMT). *In vivo*, the therapeutic efficacy of PA was evaluated using a xenograft mouse model. Transcriptomic and metabolomic profiling identified differentially expressed genes (DEGs) and metabolites, with Gene Ontology (GO) and Kyoto Encyclopedia of Genes and Genomes (KEGG) pathway enrichment analyses further exploring PA's mechanistic impact.

**Results:**

PA significantly reduced the viability, migration, invasion, clonogenic potential, and EMT of EC cells, while inducing cell-cycle arrest and apoptosis. In xenograft models, PA effectively suppressed tumor growth. Mechanistically, transcriptomic and metabolomic analyses, together with changes in ferroptosis-related markers, indicated that PA exerts its anti-cancer effects at least in part through ferroptosis activation. This conclusion was further supported by multiple ferroptosis hallmarks and ferrostatin-1 rescue, which substantially attenuated PA-induced phenotypic and biochemical alterations.

**Conclusion:**

PA suppressed EC progression by inducing ferroptosis, providing novel mechanistic insights into PA's anti-cancer properties and underscoring its potential as a therapeutic candidate for EC treatment.

## Highlights

• PA reduced the viability, migration, invasion, clonogenic potential, and EMT of EC cells, while inducing cell cycle arrest and apoptosis.
• In xenograft models, PA effectively suppressed tumor growth.
• PA exerts its anti-cancer effects primarily through the activation of ferroptosis.

## 1 Introduction

Endometrial cancer (EC) originates from the endometrial lining of the uterus and has become increasingly prevalent globally [1]. As a heterogeneous malignancy, EC is classified into two primary categories: Type I and Type II. Type I EC is predominantly estrogen-driven, typically characterized by low-grade cells and a relatively favorable prognosis. In contrast, Type II EC is estrogen-independent, often composed of high-grade cells, and is associated with a more aggressive clinical course and poorer prognosis [1]. Over the past three decades, the incidence of EC has risen by 132%, making it the sixth most common cancer among women worldwide [2]. In 2020, more than 417,000 new cases of EC were diagnosed globally, resulting in 97,370 disease-related deaths. This increasing burden is largely attributed to the aging population and the rising global prevalence of obesity and diabetes [2].

Given its strong association with modifiable risk factors, including unopposed estrogen, insulin resistance, and chronic inflammation, EC is a disease well-suited for primary prevention strategies [2]. Early diagnosis remains a critical determinant of survival in EC. However, traditional diagnostic methods, such as invasive endometrial biopsies, often limit their clinical applicability. Recent advances in minimally invasive diagnostic technologies, such as protein and DNA biomarkers and cytological analyses, offer the potential to revolutionize early EC detection. These methods not only facilitate more timely diagnoses but also enable the ongoing surveillance of high-risk populations, thereby improving the survival rates of early-stage EC. In addition to its prognostic significance, the molecular classification of EC has proven to be therapeutically valuable. This approach is increasingly used to guide treatment decisions, offering a more personalized and precise management strategy. The management of advanced and recurrent EC has notably evolved with the introduction of debulking surgery and the growing use of targeted therapies, especially immunotherapy. Immunotherapy, particularly for patients with mismatch repair deficiency (MMRd) or microsatellite instability-hypermutated (MSI-H) tumors [3], has transformed the treatment landscape, providing new therapeutic options for these patients. However, despite these advancements, the management of advanced-stage EC remains challenging, with higher recurrence rates and limited efficacy of current adjuvant therapies. The 5-year survival rates of patients with stage III/IV tumors remain at 48% and 15% [4]. Even worse, the median overall survival (OS) of recurrent/metastatic EC patients ranges from 12 to 15 months [4]. Thus, there remains a critical need to develop more effective, personalized, and systematic therapeutic approaches to combat EC progression. Such strategies not only aim to improve survival outcomes but also seek to minimize the adverse effects associated with conventional treatments, ultimately enhancing the quality of life for patients.

Despite advances in understanding the genetic basis of EC, recent research highlights the role of metabolic reprogramming in its development and progression [5]. As a hallmark of cancer, metabolic reprogramming has been recognized to regulate the division of tumor cells in nutrient-depleted conditions, affect tumor immunity, and promote metastasis [6]. Over 50% of EC cases are associated with metabolic disorders such as obesity, dyslipidemia, and altered lipid metabolism [4]. Obesity contributes to a proinflammatory microenvironment and excess estrogen production, both of which increase EC risk [7]. Dyslipidemia also acts as an independent predictor of EC, promoting tumorigenesis through metabolic disturbances [8]. Altered lipid metabolism, including abnormal fatty acid and cholesterol uptake, aberrant de novo lipogenesis, enhanced acetyl-CoA biosynthesis, and dysregulated transcriptional control of lipid metabolic genes, supports carcinogenesis, invasiveness, metastasis, and therapy resistance [4].

Considering the pivotal role of metabolic reprogramming in the occurrence, invasion, metastasis, and recurrence of EC, targeting key enzymes and transcription factors involved in this process has emerged as a promising therapeutic strategy to inhibit EC progression. Several agents, such as GW501516 (targeting PPAR), BF175 (targeting SREBP), Rosuvastatin (targeting HMGCR), A939572 (targeting SCD1), and Orlistat (targeting FASN), have demonstrated preclinical anti-tumor activity in EC [4]. Recent studies also suggest that combining metabolic reprogramming inhibitors with immunotherapy could enhance anti-tumor efficacy, offering significant clinical benefits in advanced solid tumors, particularly EC. This approach underscores the critical role of metabolic reprogramming as a potential therapeutic target in cancer management [4].

Palmitic acid (PA), a saturated fatty acid (SFA) abundant in the human diet, has garnered attention for its dual role in cancer progression and suppression through the modulation of metabolic reprogramming [9]. Present in animal and human tissues, plants, algae, fungi, yeast, and bacteria, PA constitutes approximately 20-30% of total fatty acids (FAs) in the human tissues and contributes 20-30 g per day in the average diet, highlighting its essential nutritional role [9,10]. PA is derived from dietary sources, such as animal fats, palm oil, and processed foods, as well as through endogenous biosynthesis via fatty acid synthase (FASN) from carbohydrates, amino acids, and other fatty acids. Compared to other saturated fatty acids, PA exhibits unique properties that enable it to modulate various cellular processes, including endoplasmic reticulum (ER) stress induction, membrane lipids remodeling, protein-membrane interactions, and post-translational modifications (PTM) [10]. Despite its potential detrimental metabolic effects, PA possesses well-documented physiological properties, such as anti-inflammatory and lipid metabolism-regulating functions [10,11]. In physiological conditions, such as fetal development or brain maturation, where placental and blood-brain barrier transport of PA is inefficient, its endogenous biosynthesis through de novo lipogenesis (DNL) becomes critical for maintaining tissue homeostasis and supporting essential biological functions [9]. However, under pathophysiological conditions and chronic nutritional imbalance, particularly insulin resistance, sustained DNL in the liver leads to excessive PA accumulation, disrupting homeostasis and contributing to dyslipidemia, hyperglycemia, ectopic fat deposition, and inflammation mediated by toll-like receptor 4 (TLR4) [9,11]. Dysregulated PA biosynthesis and homeostasis have also been implicated in various diseases including atherosclerosis and neurodegenerative disorders [11].

Beyond metabolic disorders, PA homeostasis significantly influences tumor progression through mechanisms such as metabolic reprogramming, inflammatory pathways, and cellular signaling. For instance, PA promotes cell proliferation, invasion, and metastasis in oral and prostate cancer by modulating key signaling pathways [12]. Conversely, extensive *in vivo* and *in vitro* studies have elucidated the anti-tumor mechanisms of PA, demonstrating its ability to induce apoptosis, inhibit cancer cell proliferation, reduce cell migration in various cancers, enhance sensitivity to chemotherapy, and improve immune function, including gastric, liver, cervical, neuroblastoma, breast, and colorectal cancers [10,13,14].

Although PA has been implicated in ferroptosis regulation in several biological contexts, the EC-specific anti-tumor mechanism and the accompanying metabolic remodeling remain insufficiently defined. Here, we systematically evaluated the anti-cancer efficacy of PA in EC using comprehensive *in vitro* phenotyping and a xenograft mouse model. We further integrated transcriptomic and metabolomic profiling to delineate coordinated pathway perturbations induced by PA and to link PA exposure with metabolic reprogramming. Importantly, we performed multi-dimensional ferroptosis validation and pharmacological rescue using ferrostatin-1 (Fer-1, a specific ferroptosis inhibitor) to substantiate that ferroptosis is mechanistically relevant and functionally required for PA-mediated EC suppression. Our findings aim to provide novel insights into the dual role of PA in cancer and its therapeutic potential in targeting metabolic reprogramming in EC.

## 2 Materials and methods

### 2.1 Chemicals

Unless otherwise specified, all chemicals used in this study were purchased from Sigma-Aldrich (Shanghai, China) and Thermo Fisher Scientific (Shanghai, China).

### 2.2 Cell culture

Ishikawa cells, authenticated by short tandem repeat (STR) profiling, were obtained from the Cell Bank of Procell Life Science & Technology (Wuhan, China). After thawing, cells were cultured in Dulbecco's Modified Eagle Medium/F12 (DMEM/F12; Biological Industries, Shanghai, China), supplemented with 10% fetal bovine serum (FBS; 10091148, Thermo Fisher Scientific, Shanghai, China) and 100 U/mL penicillin-streptomycin (P/S; 15070063, Thermo Fisher Scientific, Shanghai, China). Cells were maintained in 100-mm culture dishes (Corning, Beijing, China) in a humidified atmosphere of 5% CO_2_ at 37 °C.

### 2.3 PA treatment and cell proliferation assessment

The cytotoxicity effect of PA on EC progression was analyzed by the 3-(4,5-dimethylthiazol-2-yl)-2,5-diphenyl tetrazolium bromide (MTT) assay [15]. Ishikawa cells were seeded at a density of 5000 cells per well in 96-well plates and incubated for 24 h. Following incubation, the culture medium was supplemented with PA at concentrations of 50, 100, 200, and 400 μM (P5585, Sigma Aldrich, Shanghai, China) and incubated for an additional 24 h [13]. Negative control (NC) cells were treated with the same volume of Dulbecco's phosphate-buffered saline (DPBS; Thermo Fisher Scientific, Shanghai, China). After treatments, 10 μL of MTT solution (5 mg/mL, C0009S, Beyotime, Shanghai, China) was added to each well, and cells were incubated at 37 °C for 2 h. The formazan product was solubilized with 100 μL dimethyl sulfoxide (DMSO; Coolaber, Beijing, China), and absorbance was measured at 570 nm using a microplate reader. The inhibition rate (IR) of cell proliferation was calculated.

Cell proliferation was further confirmed using the lactate dehydrogenase (LDH) assay kit (C0017, Beyotime, Shanghai, China). Supernatants were collected after 24 h of PA treatment, and LDH release was measured according to the manufacturer's instructions, with absorbance recorded at 490 nm. According to the combined results of the MTT and LDH assessment, the PA concentration applied for the following assessments was optimized.

### 2.4 EdU staining

The impact of PA treatment on cell proliferation was assessed by 5-ethynyl-2′-deoxyuridine (EdU) incorporation, using an EdU staining kit (C0071S, Beyotime, Shanghai, China) [16]. Ishikawa cells were treated with PA for 24 h, washed with DPBS, and incubated with EdU working solution for 2 h in the dark. Cells were then stained with 4′,6-diamidino-2-phenylindole solution (DAPI, C0065, Solarbio, Beijing, China) for 5 min at 37 °C. EdU incorporation was observed using fluorescence microscopy, and relative EdU staining intensity was quantified using ImageJ software.

### 2.5 Wound healing assay

Cell migration following PA treatment was assessed using a wound-healing assay [16]. Ishikawa cells were seeded at a density of 5 × 10^4^ cells per well in 6-well plates and cultured for 24 h. A wound was created by scratching the monolayer with a 200 μL pipette tip. After removing non-adherent cells by washing with DPBS, cells were treated with culture medium containing PA or DPBS for 24 h. The wound closure was monitored by photographing at 0 and 24 h post-scratching.

### 2.6 Invasion assay

Cell invasion ability was assessed using a Transwell invasion assay [16]. Ishikawa cells (2 × 10^3^ cells per well) were resuspended in serum-free medium and plated in the upper chamber of Transwell inserts, with PA or DPBS in the culture medium. After 24 h of incubation, non-invading cells were removed, and the invading cells were fixed with methanol, stained with 0.1% crystal violet (C0121, Beyotime, Shanghai, China), and photographed. The number of invading cells was quantified using ImageJ software.

### 2.7 Clonogenicity assay

The effect of PA treatment on cell clonogenicity was assessed by plating 1 × 10^3^ Ishikawa cells in 6-well plates, and then cultured for 7 days in the presence of PA or DPBS [16]. Medium was replaced daily. Colonies were fixed with 4% paraformaldehyde (PFA; P1110, Solarbio, Beijing, China), stained with 0.1% crystal violet, and colonies were counted under a microscope.

### 2.8 Adhesion assay

Cell adhesion was evaluated by plating 5 × 10^3^ Ishikawa cells per well onto fibronectin-coated 96-well plates [16]. After 30 min of incubation at 37 °C, unadhered cells were removed by washing with DPBS, and adherent cells were fixed with methanol and stained with 0.1% crystal violet. Adherent cells were visualized under a microscope, and the number of adherent cells was quantified.

### 2.9 Cell cycle assay

The effect of PA on the cell cycle was analyzed by flow cytometry [16]. Ishikawa cells were fixed in ice-cold 70% ethanol overnight at −20 °C. Following fixation, cells were stained with a commercial Cell Cycle Analysis Kit (MA0334, Meilunbio, Dalian, China) and analyzed using a flow cytometer (BD Biosciences, San Jose, CA, USA).

### 2.10 Annexin-V staining

Apoptosis was assessed using Annexin-V FITC staining [16]. Ishikawa cells were washed with cold DPBS and incubated with an Annexin V-FITC solution (CA1020, Solarbio, Beijing, China) at room temperature according to the manufacturer's instructions. Propidium iodide (PI) was added for counterstaining, and apoptosis was analyzed by flow cytometry within 1 h.

### 2.11 Animals and In vivo xenograft model

All animal experiments were conducted in accordance with the ARRIVE guidelines and adhered to the Guidance on the Operation of the Animals (Scientific Procedures) Act 1986 and related regulations. The study protocols were approved by the Animal Care and Use Committee of Baotou Medical College (Approval ID: IMPH/202024086). The influence of sex on study outcomes was not evaluated, as only female mice were included in the experiments.

Ten female BALB/c nude mice (5 weeks old) were obtained from SPF (Beijing) Biotechnology Co., Ltd. The animals were housed in a specific pathogen-free facility under controlled environmental conditions (humidity: 60 ± 5%, temperature: 20-23 °C, 12-h light/dark cycle).

For xenograft tumor establishment, 5 × 10^5^ Ishikawa cells were subcutaneously injected into the right flank of each mouse. When the tumors reached approximately 100 mm^3^, the mice were randomly assigned to either the negative control (NC) group or the PA group (n = 5 per group). Mice in the PA group were administered subcutaneous injections of 50 mg/kg PA every two days [17], while the NC group received an equivalent volume of saline. Tumor volumes were measured daily using calipers. After 15 days of treatment, mice were euthanized via CO_2_ inhalation followed by cervical dislocation. Tumors were surgically excised, weighed, photographed, and the terminal tumor volumes were measured along the maximum axis (L) and the right-angle diameter to that axis (W) and were calculated based on the equation (length × width^2^)/2 [18].

### 2.12 HE staining of xenograft tumors

Post-excision, xenograft tumors were fixed overnight in neutral-buffered formalin, then embedded in paraffin according to standard departmental protocols. Serial sections (5 μm) were prepared and subjected to H&E staining using a commercial kit (G1120, Solarbio, Beijing, China) following the manufacturer's instructions [16].

### 2.13 Terminal deoxynucleotidyl transferase-mediated dUTP nick end-labeling (Tunnel) staining of xenograft tumors

For Tunnel staining, paraffin-embedded xenograft tumor sections underwent dehydration, paraffin embedding, sectioning, deparaffinization, and rehydration as previously described. The sections were pretreated with Protease K solution at 37 °C for 5 min, as per the manufacturer's protocol (Servicebio, G1502, Wuhan, China). Following equilibration at 37 °C for 30 min, sections were incubated with Terminal Deoxynucleotidyl Transferase (TdT) working reagent at 37 °C for 1 h. After incubation, sections were incubated with TdT buffer at room temperature for 30 min. Nuclei were counterstained with DAPI (C0065, Solarbio, Beijing, China) for 10 min in the dark. Sections were mounted with an anti-fade medium (S2100, Solarbio, Beijing, China) and subjected to microscopic analysis to visualize Tunnel-positive (apoptotic) cells. Apoptosis rates were quantified using ImageJ software [16].

### 2.14 Immunofluorescence (IF) staining of xenograft tumors

For immunofluorescence staining, paraffin-embedded tumor sections underwent dehydration, embedding, sectioning, deparaffinization, rehydration, and epitope retrieval according to departmental protocols with minor modifications [16]. Non-specific binding was blocked using 10% bovine serum albumin (BSA, SW3015, Solarbio, Beijing, China) at 37 °C for 30 min. Sections were then incubated overnight at 4 °C with primary antibodies targeting key markers of proliferation (KI-67), apoptosis (BAX and BCL-2), angiogenesis (VEGF), and metastasis (COX-2). After extensive washing in PBS, the sections were incubated with corresponding secondary antibodies in the dark at room temperature for 30 min. Nuclei were counterstained with DAPI solution, and sections were examined using fluorescence microscopy. Positive fluorescence intensities were quantified using ImageJ software. The antibodies used in this study are listed in Supplementary Table 1.

### 2.15 Transcriptomic analysis

Total RNA was extracted from PA treated Ishikawa cells using the TRIzol reagent (Invitrogen, Carlsbad, CA, USA). RNA quality was assessed using an Agilent 2100 Bioanalyzer (Agilent Technologies, Santa Clara, CA, USA) and verified by RNase-free agarose gel electrophoresis. mRNA was enriched using Oligo (dT) beads, fragmented, and reverse transcribed into cDNA using the NEBNext® Ultra™ RNA Library Prep Kit for Illumina® (New England Biolabs, Ipswich, MA, USA). The cDNA library was sequenced on an Illumina NovaSeq 6000 platform. Bioinformatics analyses were performed using HISAT2 for alignment, RSEM for transcript expression quantification, and DESeq2 for differential expression analysis. Genes with a false discovery rate (FDR) < 0.05 and absolute fold change (FC) ≥ 2 were considered differentially expressed. Gene ontology (GO) and Kyoto Encyclopedia of Genes and Genomes (KEGG) pathway enrichment analyses were performed [16,19].

### 2.16 Metabolomics analysis

Metabolites were extracted from Ishikawa cells post-treatment using standard procedures and analyzed by quadrupole time-of-flight mass spectrometry coupled with hydrophilic interaction chromatography (Applied Protein Technology Co., Ltd., Shanghai, China). Peak intensities were normalized, and the metabolite identification was performed by matching peaks with mzCloud, mzVault, and MassList databases. Differential metabolites were identified based on variable importance in projection (VIP) ≥ 1, |log2 fold change| ≥ 1, and *P < *0.05. KEGG pathway enrichment was performed using Fisher's exact test.

### 2.17 Ferroptosis assessment

To verify the accuracy of metabolomic and transcriptomic results, the potential effect of PA treatment on triggering the ferroptosis of EC cells was analyzed by assessing the ROS production, mitochondrial functions, redox imbalance, and DNA damage of Ishikawa cells. In addition, TEM analysis was performed to examine mitochondrial ultrastructure.

To analyze the ROS production, a commercial dichlorofluorescein diacetate (DCFH-DA) staining kit (S0033, Beyotime, Shanghai, China) was applied [16]. Accordingly, Ishikawa cells were washed with DPBS and incubated with 10 μM DCFH-DA solutions at 37 °C for 30 min. Following DAPI counterstaining, fluorescence intensity was quantified using ImageJ.

MitoTracker staining, JC-1 staining, and TEM were utilized to assess the mitochondrial dysfunction [20]. For MitoTracker staining, Ishikawa cells were washed with DPBS solution and incubated with 200 nM MitoTracker staining solution (C1049, Beyotime, Shanghai, China) at 37 °C for 30 min. After DAPI counterstaining, the MitoTracker staining was recorded with the MitoTracker staining intensity analyzed by ImageJ software. For JC-1 staining, Ishikawa cells were incubated with 10 μM JC-1 solutions (C2006, Beyotime, Shanghai, China) at 37 °C for 20 min, followed by the observation under a fluorescence microscope and JC-1 fluorescence intensity was quantified by ImageJ software. For TEM, Ishikawa cells were fixed with 2.5% glutaraldehyde solution for 24 h. After postfixing with 1% OsO_4_ solution, cell samples were dehydrated in graded ethanol, embedded in resin, and sectioned into ultrathin slices. After staining with uranyl acetate and lead citrate, these ultrathin sections were visualized by TEM.

For analyzing the PA-induced ferroptosis, GSH, MDA, and iron levels were measured using commercial kits (S0052 for GSH, S0131 for MDA, Beyotime, Shanghai, China, BC5315 for iron contents, Solarbio, Beijing, China) [21].

Additionally, DNA damages in Ishikawa cells were confirmed by IF staining of γH2A. Ishikawa cells were washed with DPBS solution, fixed by 4 % PFA for 15 min, and permeabilized by 0.5% Triton X-100 solution (T8200, Solarbio, Beijing, China) for 15 min. After BSA incubation, Ishikawa cells were incubated with a rabbit anti-γH2A antibody (at a 1:200 dilution in DPBS solution, ab81299, Abcam, Shanghai, China) overnight at 4 °C. After incubation, cells were incubated with a secondary antibody for 1 h. Then, the cells were incubated with the DAPI solution, mounted, and examined using fluorescence microscopy with the IF staining intensity of γH2A calculated by ImageJ software.

### 2.18 Western blot

For Western blot analysis of EMT-, apoptosis-, and ferroptosis-related proteins, total protein was extracted from Ishikawa cells using RIPA buffer (DE101, Transgen, Beijing, China) supplemented with protease inhibitors. Protein concentration was determined using the BCA assay (PC0020, Solarbio, Beijing, China). Equal amounts of protein (30 μg) were separated on SDS-PAGE gels (P1200, Solarbio, Beijing, China) and transferred to polyvinylidene fluoride (PVDF) membranes (PVH00010, Millipore, Beijing, China). After blocking with 5% BSA for 1 h, the membranes were incubated with primary antibodies overnight at 4 °C. After washing, membranes were incubated with secondary antibodies conjugated with HRP (1:5000; Abcam, Cambridge, MA, USA) and developed using an enhanced chemiluminescence (ECL) detection system. Protein bands were visualized and quantified using ImageJ software. The antibodies used in this study are listed in Supplementary Table 1.

### 2.19 Statistical analysis

Experimental data are presented as mean ± standard deviation (SD) and were analyzed using one-way ANOVA followed by the LSD test using SPSS software (IBM, version 19.0). *P* < 0.05 was set as significant.

## 3 Results

### 3.1 In vitro therapeutic effects of PA against EC progression

As illustrated in Supplementary Fig. 1, both MTT and LDH assays revealed robust inhibitory effects of PA on the proliferation of Ishikawa cells. A dose-dependent reduction in cell viability was observed (Supplementary Fig. 1 A, *P < *0.05), accompanied by a significant increase in LDH release (Supplementary Fig. 1 B, *P < *0.05) across all PA treatment groups relative to the NC group. Given the marked intergroup differences in viability and LDH release, a PA concentration of 200 μM was selected for subsequent analyses.

**Fig. 1:**
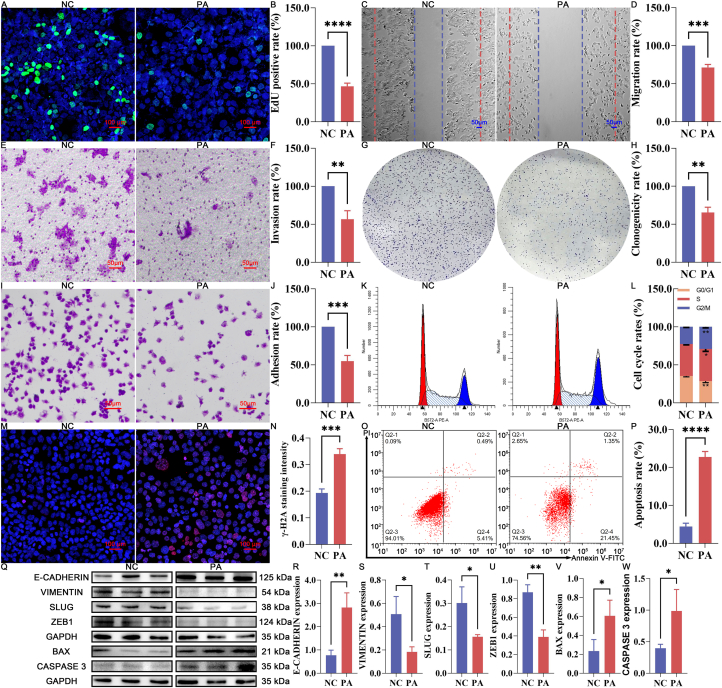
Effect of PA treatment on inhibiting the malignant behaviors of Ishikawa cells (A-B) EdU staining and quantification. (C-D) Wound-healing assay and quantification of migration. (E-F) Transwell invasion assay and quantification. (G-H) Colony formation assay and quantification. (I-J) Cell adhesion assay and quantification. (K-L) Cell-cycle distribution and quantification. (M − N) γH2A immunofluorescence staining and quantification. (O–P) Annexin V/PI apoptosis assay and quantification. (Q-W) Immunoblotting of EMT- and apoptosis-related proteins and quantification. NC represents the negative control group, while PA represents the PA-treated experimental group. ns represents *P* > 0.05 with * representing *P* < 0.05, ** representing *P* < 0.01, *** representing *P* < 0.001, and **** representing *P* < 0.0001.

Consistent with the MTT and LDH data, EdU incorporation assays demonstrated a substantial decrease in the ratio of EdU-positive cells to total DAPI-positive cells in the PA-treated group (Fig. 1A–B, *P < *0.05). Furthermore, PA treatment significantly impaired key malignant phenotypes, including cell migration (Fig. 1C–D, *P < *0.05), invasion (Fig. 1E–F, *P < *0.05), clonogenicity (Fig. 1G–H, *P < *0.05), and cell adhesion (Fig. 1I–J, *P < *0.05). Additionally, flow-cytometric cell-cycle profiling (Fig. 1K–L) showed that PA treatment significantly increased the G2/M-phase population, accompanied by decreased G0/G1-and S-phase fractions (*P* < 0.05), indicating a clear G2/M-phase cell-cycle arrest in Ishikawa cells. Consistently, PA-treated cells exhibited markedly elevated DNA damage (Fig. 1M−N, *P* < 0.05) and increased apoptosis (Fig. 1O–P, *P* < 0.05), supporting the growth-inhibitory effect of PA, which was consistent with the reduced proliferative capacity observed in EdU assays.

The molecular basis underlying these therapeutic effects was further corroborated by differential expression of EMT-related markers (E-CADHERIN, VIMENTIN, SLUG, and ZEB1) and apoptosis-related proteins (BAX and Caspase 3), which exhibited significant changes between the PA and NC groups (Fig. 1Q–W, *P < *0.05).

Collectively, these results demonstrated that PA exerts potent therapeutic effects by disrupting key processes involved in cell survival, proliferation, migration, invasion, clonogenicity, adhesion, and EMT, while simultaneously promoting significant cell-cycle arrest, DNA damage, and apoptosis.

### 3.2 In vivo therapeutic efficacy of PA in EC progression

**Fig. 2:**
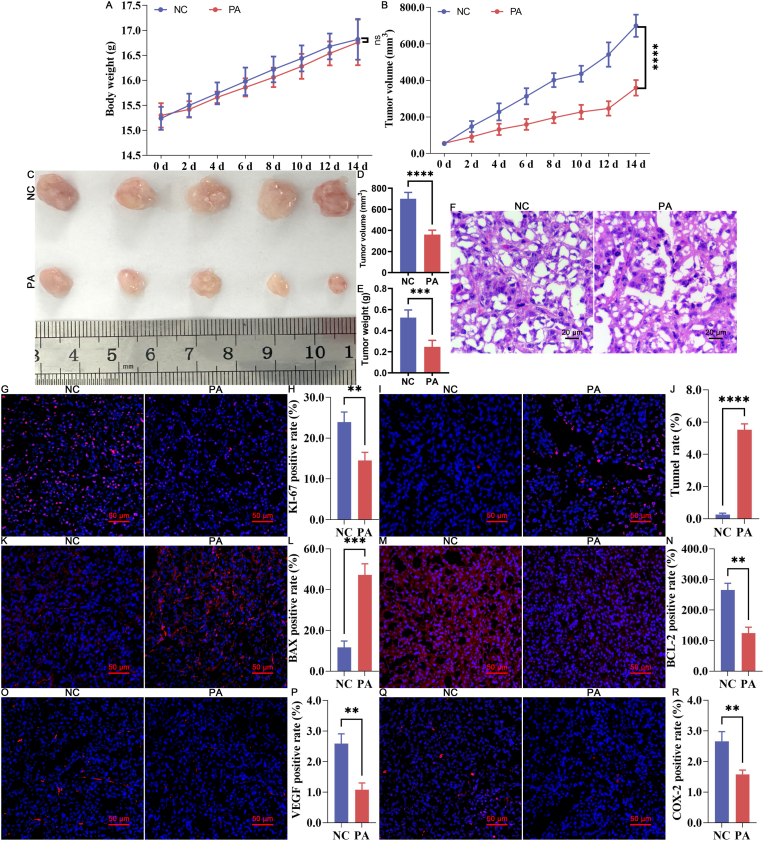
*In vivo* antitumor effect of PA treatment against EC progression (A) Body weight changes of each group. (B) Growth curve of xenograft tissues. (C) Representative morphology of xenograft tissues. (D-E) Quantitative results of tumor volume and weight. (F) Representative HE staining result of xenograft tissues. (G-H) KI-67 immunofluorescence staining and quantification. (I-J) Tunnel staining and quantification. (K-L) BAX immunofluorescence staining and quantification. (M − N) BCL-2 immunofluorescence staining and quantification. (O–P) VEGF immunofluorescence staining and quantification. (Q-R) COX-2 immunofluorescence staining and quantification. NC represents the negative control group, while PA represents the PA-treated experimental group. ns represents *P* > 0.05 with * representing *P* < 0.05, ** representing *P* < 0.01, *** representing *P* < 0.001, and **** representing *P* < 0.0001.

Building on the encouraging *in vitro* findings, the *in vivo* anti-tumor efficacy of PA was evaluated using a subcutaneous Ishikawa cell xenograft mouse model. Throughout the treatment period, there were no significant changes in the body weights of the mice across all groups (Fig. 2 A, P > 0.05). However, the PA-treated group exhibited significantly reduced tumor growth compared to the NC group (Fig. 2 B, *P < *0.05). Post-sacrifice analyses revealed substantial reductions in tumor volume (Fig. 2C&D, *P < *0.05) and weight (Fig. 2 E, *P < *0.05) in the PA-treated group.

To further substantiate the therapeutic effects of PA, histological analyses of tumor tissues were performed. H&E staining revealed significant cellular shrinkage and nuclear degradation in the PA-treated tumors, indicative of enhanced apoptosis (Fig. 2 F). In contrast, tumors treated with the vehicle control exhibited minimal signs of apoptosis or necrosis. IF staining further corroborated these findings, demonstrating a marked reduction in the expression of the proliferation marker KI-67 (Fig. 2G–H, *P < *0.05) in the PA-treated group. In addition, the Tunnel staining results confirmed a significantly higher proportion of apoptotic cells (red) in the PA-treated tumors compared to the NC group (Fig. 2I–J, *P < *0.05). Combined with the differential expression patterns of the apoptosis marker BAX (Fig. 2K–L, *P* < 0.05) and BCL-2 (Fig. 2M−N, *P* < 0.05), angiogenesis markers VEGF (Fig. 2O–P, *P* < 0.05), and metastasis marker COX-2 (Fig. 2Q–R, *P* < 0.05) between the NC and PA groups, these results demonstrated significant therapeutic efficacy of PA *in vivo*, which is associated with the suppression of tumor proliferation, metastasis, and angiogenesis, alongside the induction of apoptosis.

### 3.3 Molecular mechanisms underlying the therapeutic effects of PA in EC progression

**Fig. 3:**
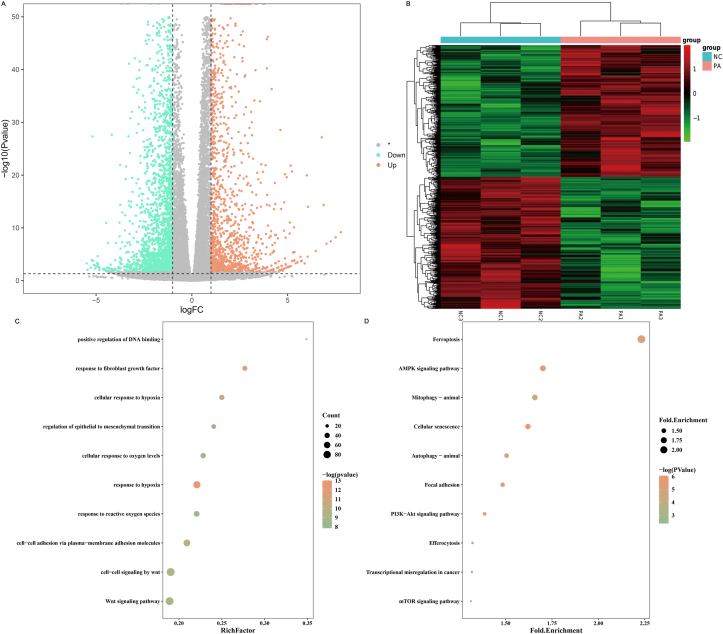
Effect of PA treatment on inducing transcriptomic alterations of Ishikawa cells (A) Volcano plots of DEGs. (B) Heat map of DEGs. (C) Biological process of GO enrichment analysis. (D) KEGG pathway enrichment analysis. NC represents the negative control group, while PA represents the PA-treated experimental group.

To further elucidate the molecular mechanisms underlying PA's therapeutic effects in EC, transcriptomic analysis was performed on Ishikawa cells exposed to PA. A total of 12,595 transcripts were identified in the NC and PA-treated groups. Differential gene expression analysis revealed that PA treatment led to the regulation of 2721 genes, with 1361 upregulated and 1360 downregulated (Fig. 3 A). Hierarchical clustering based on heatmap analysis revealed clear distinctions in gene expression profiles between the two groups (Fig. 3 B), highlighting the profound transcriptional shifts induced by PA.

GO enrichment analysis provided further insights into the biological processes affected by PA, with significant enrichment in processes such as cell-cell signaling by wnt, Wnt signaling pathway, response to hypoxia, cell-cell adhesion via plasma-membrane adhesion molecules, response to reactive oxygen species, cellular response to oxygen levels, cellular response to hypoxia, response to fibroblast growth factor, regulation of epithelial to mesenchymal transition, and positive regulation of DNA binding (Fig. 3C). Next, KEGG was performed to identify key signaling pathways modulated by PA. KEGG analysis revealed significant perturbations in pathways related to Ferroptosis, AMPK signaling pathway, Mitophagy-animal, Cellular senescence, Autophagy-animal, Focal adhesion, PI3K-Akt signaling pathway, Efferocytosis, Transcriptional misregulation in cancer, and mTOR signaling pathway (Fig. 3 D).

**Fig. 4:**
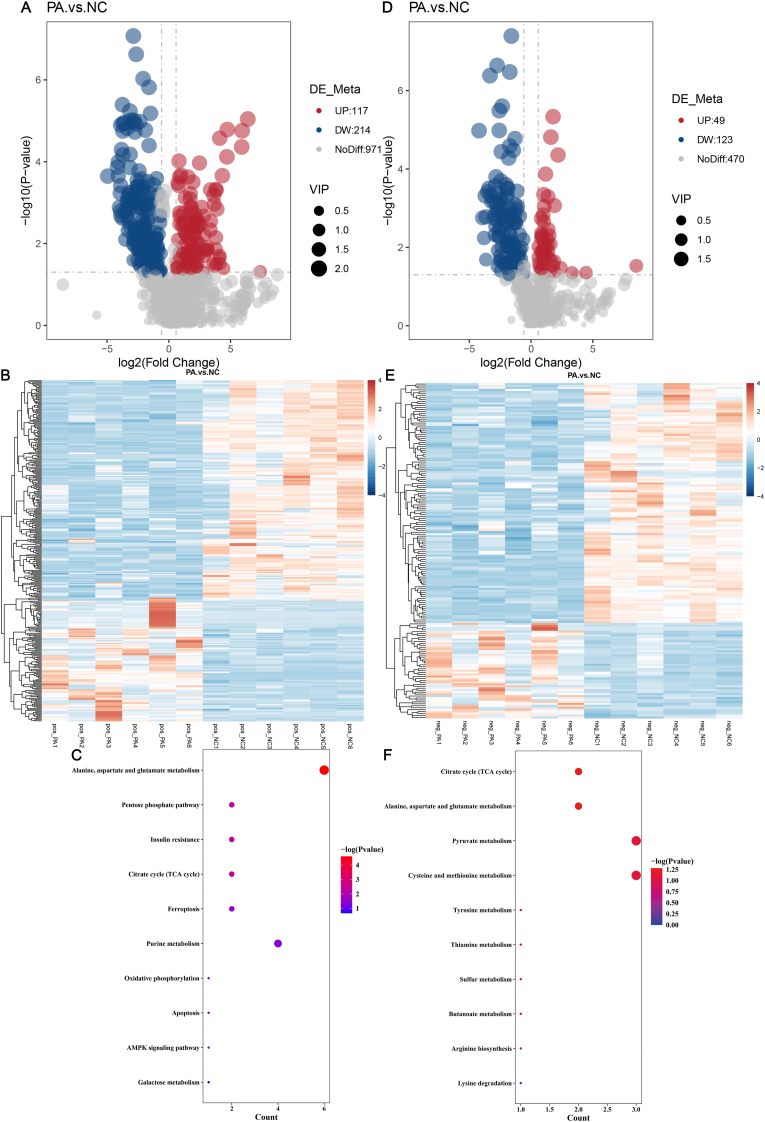
Effect of PA treatment on inducing metabolic disturbances of Ishikawa cells (A) Volcano plots of differential metabolites in positive ion mode. (B) Heat map of differential metabolites in positive ion mode. (C) KEGG pathway enrichment analysis of differential metabolites in positive ion mode. (D) Volcano plots of differential metabolites in negative ion mode. (E) Heat map of differential metabolites in negative ion mode. (F) KEGG pathway enrichment analysis of differential metabolites in negative ion mode. NC represents the negative control group, while PA represents the PA-treated experimental group.

Metabolomic analysis was further performed to investigate metabolic alterations in PA-treated EC cells. A total of 12 samples were analyzed, resulting in the identification of 1302 metabolites in positive ion mode and 642 metabolites in negative ion mode. Differential metabolites were screened based on the following criteria: variable importance in projection (VIP) > 1.0, fold change (FC) > 1.5 or FC < 0.667, and a P-value <0.05. For instance, in the PA versus NC comparison group, 331 metabolites in positive ion mode exhibited significant differences, with 117 metabolites upregulated and 214 downregulated (Fig. 4A–B). Similarly, 172 metabolites in negative ion mode showed significant differences, including 49 upregulated and 123 downregulated metabolites (Fig. 4D–E). Subsequently, all identified metabolites were systematically annotated, and pathway enrichment analysis was performed using the KEGG database. The results of the KEGG pathway enrichment analysis indicate that the metabolites identified in the positive ion mode are involved in several key metabolic and signaling pathways, including alanine, aspartate, and glutamate metabolism, the pentose phosphate pathway (PPP), insulin resistance, the tricarboxylic acid (TCA) cycle, ferroptosis, purine metabolism, oxidative phosphorylation, apoptosis, the AMPK signaling pathway, and galactose metabolism (Fig. 4C). Similarly, KEGG pathway enrichment analysis under the negative ion mode (Fig. 4 F) reveals that the identified metabolites play a crucial role in regulating the tricarboxylic acid (TCA) cycle, alanine, aspartate, and glutamate metabolism, cysteine and methionine metabolism, pyruvate metabolism, arginine biosynthesis, tyrosine metabolism, butanoate metabolism, thiamine metabolism, sulfur metabolism, and lysine degradation.

### 3.4 Therapeutic effects of PA in EC progression through ferroptosis induction

The induction of ferroptosis by PA was further validated by assessing key ferroptosis-associated markers, including intracellular ROS production, mitochondrial dysfunction, lipid peroxidation, GSH depletion, iron accumulation, and DNA damage, as well as the expression levels of ferroptosis-related proteins.

**Fig. 5:**
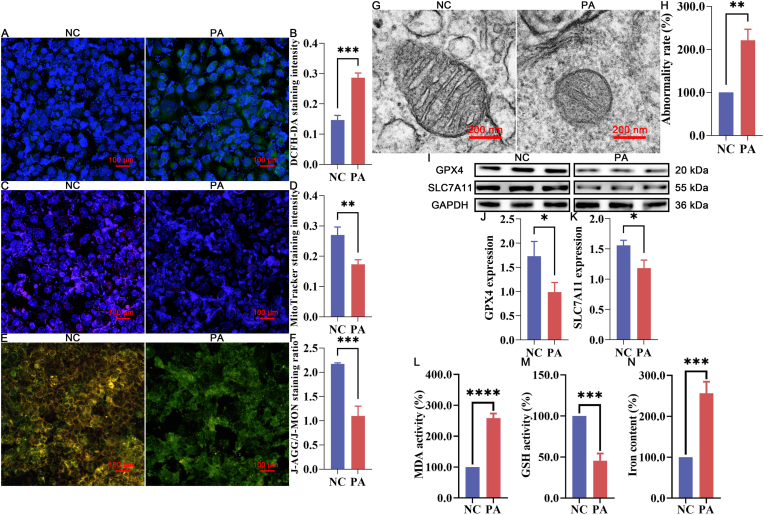
Effect of PA treatment in triggering ferroptosis of Ishikawa cells (A-B) DCFH-DA staining and quantification. (C-D) MitoTracker staining and quantification. (E-F) JC-1 staining and quantification. (G-H) Mitochondrial ultrastructural abnormalities and quantification. (I–K) Immunoblotting of ferroptosis-related proteins and quantification. (L) Quantitative results of MDA activity. (M) Quantitative results of GSH activity. (N) Quantitative results of Iron content. NC represents the negative control group, while PA represents the PA-treated experimental group. ns represents *P* > 0.05 with * representing *P* < 0.05, ** representing *P* < 0.01, *** representing *P* < 0.001, and **** representing *P* < 0.0001.

Compared to the NC group, PA treatment significantly increased ROS production, as evidenced by enhanced DCFH-DA staining intensity (Fig. 5A–B, *P < *0.05). This increase in ROS was accompanied by notable mitochondrial dysfunction, indicated by reduced MitoTracker staining intensity (Fig. 5C–D, *P < *0.05), altered mitochondrial membrane potential (ΔΨm) (Fig. 5E–F, *P < *0.05), and structural changes (Fig. 5G–H, *P < *0.05), including decreased mitochondrial volume and increased membrane density in PA-treated cells.

Furthermore, PA treatment led to significant changes in the expression of key ferroptosis-regulating proteins, SLC7A11 and GPX4 (Fig. 5I–K, *P < *0.05), confirming the activation of ferroptosis. Additional analyses revealed significant increases in lipid peroxidation, as indicated by elevated MDA levels (Fig. 5 L, *P < *0.05), and GSH depletion (Fig. 5 M, *P < *0.05), alongside increased iron content (Fig. 5 N, *P < *0.05) in the PA-treated Ishikawa cells.

In addition, Fer-1 was employed to confirm the initiation effect of PA on the ferroptosis of Ishikawa cells. Treatment with 10 μM Fer-1 not only attenuated the PA-induced alterations in cell survival (Supplementary Fig. 2), proliferation, migration, invasion, clonogenicity, adhesion, cell-cycle arrest, and apoptosis (Supplementary Fig. 3) but also reversed disturbances in the GSH depletion, MDA levels, iron accumulation, and ROS production of PA-treated Ishikawa cells (Supplementary Fig. 4). Together, these results indicate that ferroptosis is functionally required for PA-mediated suppression of EC progression.

## 4 Discussion

PA, as one of the most abundant saturated fatty acids in nature, is ubiquitously distributed across diverse biological systems, including animal tissues, human organs, plants, algae, fungi, yeast, and bacteria [9,11]. Its distribution exhibits substantial interspecies variability and intraspecies differences, influenced by environmental factors such as soil pH, nutrient ion interactions, age, hydration status, and climatic conditions, which collectively influence the PA content and metabolic dynamics [9]. As a predominant saturated fatty acid in humans, PA is derived either from dietary sources or through endogenous biosynthesis via conversion from other fatty acids, carbohydrates, and amino acids. PA constitutes approximately 20-30% of total fatty acids in membrane phospholipids (PL) and triacylglycerols (TAG), with an estimated 3.5 kg of PA present in a 70-kg adult. As a major dietary lipid, PA is highly abundant in palm oil (44% of total fat), meat and dairy products (50-60%), cocoa butter (26%), and olive oil (8-20%), while also accounting for 20-30% of total lipids in human breast milk. Despite individual variations in dietary PA consumption, the average daily intake ranges from 20 to 30 g, contributing 8-10% of total caloric intake [22]. PA homeostasis in mammalian tissues is tightly regulated, with dietary fluctuations typically exerting minimal influence on tissue PA levels due to compensatory DNL. However, under specific pathophysiological conditions, including metabolic disorders, obesity, or diabetes, DNL activation may result in PA accumulation, disrupting lipid homeostasis. The body counterbalances excess PA via δ9-desaturation into palmitoleic acid (POA) or elongation into stearic acid, which is further desaturated into oleic acid, thereby maintaining lipid metabolic equilibrium [11,23]. Compared to other saturated fatty acids, PA exhibits unique physiological functions, particularly in its roles in membrane architecture, energy metabolism, signal transduction, and PTMs. Mechanistically, PA induces endoplasmic reticulum (ER) stress by increasing phospholipid saturation, subsequently altering membrane fluidity and protein-membrane interactions [24]. These alterations activate ER stress sensors, including protein kinase RNA-like ER kinase (PERK), inositol-requiring enzyme 1 (IRE1), and activating transcription factor 6 (ATF6) [10]. Additionally, PA dynamically regulates protein palmitoylation through palmitoyl transferases (PATs), modifying protein stability, localization, and interaction, ultimately influencing multiple oncogenic signaling pathways [[25], [26], [27]].

The role of PA in cancer progression remains a subject of intense debate. Some studies suggest that PA accumulation promotes lipotoxicity, chronic inflammation, oxidative stress, and metabolic reprogramming, thereby facilitating tumorigenesis in oral carcinoma, melanoma, and pancreatic cancer [12,28]. However, accumulating evidence demonstrates that PA exerts significant anti-cancer activity in multiple cancer models [10,13,14]. The anti-cancer mechanisms of PA involve apoptosis induction (p53 activation, Bcl-2/Bax imbalance, and Caspase 3 activation), proliferation inhibition (cell-cycle arrest), oxidative stress-mediated cytotoxicity (ROS generation and mitochondrial apoptosis activation), metastasis suppression (regulation of NF-κB, STAT3, and EMT-related proteins), and chemosensitization (inhibition of PI3K/Akt pathway). A recent breakthrough study by Victoria L. Bae-Jump's team demonstrated that PA effectively suppresses EC progression [17]. However, the precise molecular mechanisms underlying PA-mediated EC inhibition remain largely undefined, necessitating further investigation. In this context, the present study advances prior observations by establishing EC-relevant anti-tumor activity of PA through systematic *in vitro* phenotyping and *in vivo* xenograft validation, and by integrating transcriptomic and metabolomic profiling to define a coordinated landscape of pathway remodeling upon PA exposure. Notably, this multi-omics framework was coupled with multi-hallmark ferroptosis assessment and Fer-1 rescue, thereby functionally validating ferroptosis as a mechanistic driver rather than inferring it from single-marker changes alone.

The current study systematically investigated the anti-cancer effects of PA on EC progression using both *in vitro* cellular models and *in vivo* xenograft models. MTT and LDH assays identified 200 μM PA as the optimal treatment concentration, effectively inducing biological responses in Ishikawa cells while minimizing cytotoxicity. Functional assays, including EdU staining, wound healing, Transwell invasion, colony formation, and cell adhesion assays, demonstrated PA significantly inhibited the proliferation, migration, invasion, clonogenicity, and adhesion of EC cells. Cell cycle analysis and apoptosis detection revealed that PA induced cell-cycle arrest and apoptosis, findings corroborated by modulations in EMT and apoptosis-related protein expression. Consistently, in xenograft mouse models, PA treatment resulted in a significant reduction in tumor volume and weight. The IF staining demonstrated decreased KI-67, VEGF, COX-2, and BCL-2 expression alongside increased BAX levels. Tunnel staining confirmed enhanced apoptosis in PA-treated tumors, reinforcing the anti-cancer efficacy of PA treatment across multiple experimental models, in agreement with Bae-Jump's findings.

To delineate the mechanistic basis of PA-mediated EC suppression, we conducted integrated transcriptomic and metabolomic analyses. Transcriptomic profiling identified 2721 DEGs enriched in key cancer-associated pathways, including ferroptosis [[29], [30], [31]], AMPK signaling [[32], [33], [34]], mitophagy [31,35,36], cellular senescence [37], autophagy [[38], [39], [40]], focal adhesion, PI3K/Akt signaling [41], efferocytosis, transcriptional dysregulation in cancer, and mTOR pathways [[42], [43], [44]]. While some enriched pathways have been previously associated with PA interactions, many have not been previously linked to PA in EC, providing critical insights into the cancer-inhibitory effects of PA interactions. Metabolomic profiling further revealed 1302 positive-ion and 642 negative-ion metabolites, with differential metabolites enriched in alanine/aspartate/glutamate metabolism, pentose phosphate pathway, insulin resistance, TCA cycle, ferroptosis, purine metabolism, oxidative phosphorylation, apoptosis, AMPK signaling, galactose metabolism, cysteine/methionine metabolism, pyruvate metabolism, arginine biosynthesis, tyrosine metabolism, butanoate metabolism, thiamine metabolism, sulfur metabolism, and lysine degradation. Importantly, these pathway-level metabolic signatures offered mechanistic insight consistent with PA-triggered metabolic reprogramming and the cellular biochemical prerequisites for ferroptosis, particularly through redox buffering capacity, mitochondrial ROS pressure, and iron-dependent lipid oxidation.

To strengthen the mechanistic link between these metabolomic signatures and ferroptosis, the enriched pathways can be interpreted as converging on three ferroptosis-defining biochemical axes that regulate glutathione availability, iron handling, and lipid peroxidation propagation.

First, amino-acid pathway enrichment, prominently alanine, aspartate and glutamate metabolism together with cysteine and methionine metabolism and sulfur metabolism, is closely aligned with redox buffering capacity because these networks influence cysteine supply and glutathione biosynthesis. Cysteine availability is rate-limiting for GSH generation, and SLC7A11 governs cystine import to sustain intracellular cysteine pools. Therefore, remodeling of these amino-acid networks is mechanistically consistent with reduced GSH resilience and impaired GPX4-dependent detoxification of lipid hydroperoxides, thereby increasing susceptibility to lipid peroxidation and ferroptotic cell death. Second, pentose phosphate pathway enrichment provides a direct metabolic connection to the antioxidant arm of ferroptosis control through NADPH supply. NADPH is required to maintain GSH in its reduced form and to constrain oxidative chain reactions. Thus, altered PPP activity is consistent with reduced cellular reducing capacity, accelerated oxidative stress propagation, and a greater propensity for lipid peroxide accumulation under ferroptotic stress. Third, enrichment of pyruvate metabolism, the TCA cycle, and oxidative phosphorylation indicates mitochondrial and bioenergetic remodeling. Such remodeling can elevate ROS pressure and promote conditions that accelerate membrane lipid oxidation. In parallel, ferroptosis requires a permissive iron state because iron catalyzes oxidative reactions that drive lipid peroxide formation. While metabolomics does not directly quantify iron species, the concurrent experimental evidence of increased iron content, together with transcriptomic enrichment of ferroptosis-associated programs, supports the interpretation that PA-driven metabolic remodeling occurs in a cellular context where iron-dependent lipid peroxidation can be amplified.

Taken together, these multi-omics signatures support a coherent model in which PA induces metabolic reprogramming that compromises redox buffering, reshapes mitochondrial energy metabolism, and creates conditions permissive for iron-dependent lipid peroxidation, thereby predisposing EC cells to ferroptosis. Through integrated transcriptomic and metabolomic analyses, the current study reveals that PA suppressed EC progression by orchestrating metabolic reprogramming to induce ferroptosis, an iron-dependent form of programmed cell death, characterized by lipid peroxidation and ROS accumulation. Unlike classical cell death modalities (apoptosis, necrosis, and autophagy), ferroptosis exhibits distinct morphological and molecular features and has gained prominence in oncology, particularly as a potential strategy to overcome chemoresistance [[45], [46], [47]]. Our findings demonstrated that PA treatment significantly elevated intracellular ROS levels, induced mitochondrial dysfunction (manifested as membrane potential collapse, cristae fragmentation, and condensation), increased MDA and free iron levels, depleted GSH, and downregulated ferroptosis-inhibitory proteins GPX4 and SLC7A11 in Ishikawa cells - all hallmarks of ferroptosis. To confirm ferroptosis as the central mechanism underlying PA-mediated EC suppression, we employed the ferroptosis-specific inhibitor Fer-1 [[48], [49], [50]], which effectively reversed PA-induced apoptosis, restored cellular viability and proliferation, reduced ROS/MDA/free iron accumulation, and partially rescued GSH levels. These results strongly support ferroptosis as a pivotal pathway mediating the antitumor effect of PA against EC progression.

Importantly, the integration of pathway-level multi-omics remodeling with multi-hallmark ferroptosis readouts and Fer-1 rescue supports the mechanistic conclusion that PA-associated metabolic reprogramming is functionally aligned with ferroptosis execution, rather than reflecting nonspecific cytotoxicity alone. Accordingly, the innovation of the present work lies not in observing GPX4 or SLC7A11 changes in isolation, but in establishing an EC-specific, multi-level mechanistic framework that connects PA-driven metabolic remodeling to ferroptosis-defining biochemical determinants and validates ferroptosis as a functional driver of the anti-tumor phenotype.

Recent studies have elucidated the ferroptosis-inducing capacity of PA across disease models. Wang et al. identified PA-mediated downregulation of GPX4 and heat shock factor 1 (HSF1), leading to cardiomyocyte ferroptosis [30]. Zhou et al. demonstrated that PA-induced activation of CD36 promotes ferroptosis in colorectal cancer cells, suppressing tumor progression [29]. Zhu et al. found that PA enhances ceramide synthesis while inhibiting GPX4, driving ferroptosis in pancreatic β-cells [51]. Similarly, Liao et al. uncovered Cytochrome P450-2E1 (CYP2E1) upregulation and Sterol regulatory element binding protein 1 (SREBP1) nuclear translocation as key drivers of PA-induced ferroptosis in hepatocytes, contributing to metabolic dysfunction-associated fatty liver disease (MAFLD) [52]. Furthermore, Guan et al. revealed PA-mediated downregulation of GPX4 and SLC7A11 in vascular endothelial cells, a finding corroborated by Xie et al. [53,54]. Collectively, these studies establish PA as a universal ferroptosis inducer across diverse cell types, highlighting its therapeutic potential in oncology.

As a central node for intracellular free fatty acid metabolism, PA exerts its biological effects through three primary metabolic pathways. Firstly, PA undergoes mitochondrial β-oxidation to generate ATP, sustaining cellular energy metabolism. Secondly, PA serves as a substrate for phospholipid and triacylglycerol biosynthesis, influencing membrane architecture and signal transduction. Lastly, PA is converted to monounsaturated fatty acids (MUFAs) via fatty acid desaturases (FADS). Notably, PA is not directly converted into polyunsaturated fatty acids (PUFAs). However, Zhang et al. demonstrated that PA activates peroxisome proliferator-activated receptor α (PPARα), leading to upregulation of fatty acid desaturase 1/2 (FADS1/2) and elongation of very-long-chain fatty acid proteins 2/5 (ELOVL2/5). This cascade facilitates the desaturation and elongation of α-linoleic acid (LA) and α-linolenic acid (ALA), ultimately generating PUFAs such as arachidonic acid (AA) [55]. Under oxidative stress and Fenton reaction conditions, PUFAs undergo lipid peroxidation, producing lipid peroxides like 4-hydroxynonenal (4-HNE) that trigger ferroptosis. Additionally, PA is converted to lysophosphatidic acid (LPA) by glycerol-3-phosphate acyltransferase 1 (GPAT1), which then generates phosphatidic acid. This metabolite activates protein kinase C ζ (PKC ζ), modulating the activity of phosphatidyl ethanolamine-binding protein 1 (PEBP1) and 15-lipoxygenase (15-LO) complex, further amplifying PUFA peroxidation and ferroptosis induction [55].

Several limitations of this study should be acknowledged. First, the *in vitro* mechanistic investigations were primarily conducted in a single EC cell line (Ishikawa). Although the anti-tumor efficacy of PA was corroborated *in vivo* using a xenograft model and supported by multi-dimensional ferroptosis hallmarks together with Fer-1 rescue, validation in additional EC cell lines, particularly more aggressive or Type II models, will be important to strengthen the generalizability of our conclusions. Second, while integrated transcriptomic and metabolomic profiling revealed coordinated pathway perturbations consistent with metabolic reprogramming and ferroptosis activation, the metabolomic evidence remains largely associative at the metabolite level. Future studies should further interrogate the causal contribution of key metabolic nodes identified by KEGG enrichment to core ferroptosis determinants, including cysteine/GSH availability, iron handling, and lipid peroxidation dynamics, using targeted metabolomics, metabolic flux analyses, and genetic or pharmacological manipulation of relevant enzymes and transporters. Finally, given the breadth of experimental outputs, continued efforts to refine mechanistic boundaries and improve clarity of presentation will facilitate interpretation and translation of these findings.

Despite these limitations, our findings support PA as a ferroptosis-inducing agent with potential anti-cancer relevance in EC. While the precise contribution and hierarchy of the metabolically regulated processes driving PA-induced ferroptosis warrant further investigation, the current multi-omics framework together with functional ferroptosis validation provides a strong foundation for future work. Future studies will be important to dissect the interaction between PA and ferroptosis-associated metabolic networks in additional EC models and to evaluate its clinical relevance as a ferroptosis-targeting therapeutic candidate in EC and beyond.

## CRediT authorship contribution statement

Shuting Lan: Writing-original draft, Validation, Investigation, Formal analysis, Funding acquisition. Xiaomei Sun: Writing original draft, Validation, Investigation, Formal analysis. Qiyuan Bo: Writing review & editing, Formal analysis. Suriguga Wang: Writing review & editing, Methodology. Yifan Qin: Writing review & editing, Methodology. Jianying Mao: Writing review & editing, Project administration, Conceptualization, Funding acquisition.

## Declaration of competing interest

None.

## Data Availability

Data will be made available on request.
