# Supplementary material for: Anti-cancer effect of palmitic acid against the endometrial cancer progression via inducing ferroptosis
Source: Biochem Biophys Rep. 2026 Feb 10;45:102499. doi: 10.1016/j.bbrep.2026.102499 (PMC12914859; doi:10.1016/j.bbrep.2026.102499)

**Supplementation Table and Figures legends**

**Supplementation Tab. 1 Antibody applied in the present study**

**Supplementation Fig. 1 Effect of PA on the viability of Ishikawa cells**

(A) Representative micrographs of Ishikawa cells following 24 h of treatment with varying concentrations of PA. (B) Quantitative viability of Ishikawa cells following 24 h of treatment with varying concentrations of PA. (C) Quantification of LDH release from Ishikawa cells following 24 h of treatment with varying concentrations of PA. NC represents the negative control group, while PA represents the PA-treated experimental group.

**Supplementation Fig. 2** **Effect of Fer-1 intervention on PA-induced inhibition of Ishikawa cell viability**

NC represents the negative control group. PA represents the PA-treated experimental group. Fer-1 represents the Fer-1 treatment alone. PA+Fer-1 represents the co-treatment of PA and Fer-1. ns represents *P* > 0.05 with * representing *P* < 0.05, ** representing *P* < 0.01, *** representing *P* < 0.001, and **** representing *P* < 0.0001.

**Supplementation Fig.3 Effect of Fer-1 intervention on the inhibited malignant behaviors of Ishikawa cells induced by PA treatments**

(A-B) Representative and quantitative result of cell migration. (C-D) Representative and quantitative result of cell invasion. (E-F) Representative and quantitative result of clonogenicity assay. (G-H) Representative and quantitative result of cell adhesion. (I-J) Representative and quantitative of cell cycle distribution. (K-L) Representative and quantitative result of Annexin V/PI staining. NC represents the negative control group. PA represents the PA-treated experimental group. Fer-1 represents the Fer-1 treatment alone. PA+Fer-1 represents the co-treatment of PA and Fer-1. ns represents *P* > 0.05 with * representing *P* < 0.05, ** representing *P* < 0.01, *** representing *P* < 0.001, and **** representing *P* < 0.0001.

**Supplementation Fig.4 Effect of Fer-1 intervention on the ferroptosis of Ishikawa cells induced by PA treatments**

(A-B) Representative and quantitative result of DCFH-DA staining. (C) Quantitative results of MDA activity. (D) Quantitative results of GSH activity. (E) Quantitative results of Iron content. NC represents the negative control group. PA represents the PA-treated experimental group. Fer-1 represents the Fer-1 treatment alone. PA+Fer-1 represents the co-treatment of PA and Fer-1. ns represents *P* > 0.05 with * representing *P* < 0.05, ** representing *P* < 0.01, *** representing *P* < 0.001, and **** representing *P* < 0.0001.

**Supplementation Tab.1 Antibody applied in the present study**

| **Primary antibodies for Western blot** | | | | |
| --- | --- | --- | --- | --- |
| **Antibody** | **Catalog number** | **Source** | **Dilution** | **Company** |
| E-CADHERIN | AF0138 | Mouse | 1:1000 | Beyotime |
| VIMENTIN | AF0318 | Mouse | 1:1000 | Beyotime |
| SLUG | AF7998 | Rabbit | 1:1000 | Beyotime |
| ZEB1 | AF8388 | Rabbit | 1:1000 | Beyotime |
| BAX | AF0057 | Rabbit | 1:1000 | Beyotime |
| CASPASE 3 | AF0081 | Rabbit | 1:1000 | Beyotime |
| SLC7A11 | AF7992 | Rabbit | 1:1000 | Beyotime |
| GPX4 | AF7020 | Rabbit | 1:1000 | Beyotime |
| GAPDH | AF2819 | Mouse | 1:2000 | Beyotime |
| **Primary antibodies for IF staining** | | | | |
| **Antibodies** | **Catalog number** | **Source** | **Dilution** | **Company** |
| KI-67 | GB111499 | Rabbit | 1:500 | Servicebio |
| BAX | GB154122 | Rabbit | 1:500 | Servicebio |
| BCL-2 | GB154380 | Rabbit | 1:500 | Servicebio |
| VEGF | GB15165 | Rabbit | 1:500 | Servicebio |
| COX2 | GB11077-1 | Rabbit | 1:200 | Servicebio |
| **Secondary antibodies for Western blot** | | | | |
| **Antibodies** | **Catalog number** | **Source** | **Dilution** | **Company** |
| HRP-conjugated Affinipure Goat Anti-Mouse IgG | SA00001-1 | Goat | 1:10000 | Proteintech |
| Goat Anti-Rabbit IgG H&L HPR | S0001 | Goat | 1:10000 | Affinity Biosciences |
| **Secondary antibodies for IF staining** | | | | |
| **Antibodies** | **Catalog number** | **Source** | **Dilution** | **Company** |
| CY3-labeled Goat Anti-Rabbit IgG | GB21303 | Goat | 1:300 | Servicebio |

**Supplementation Fig. 1 Effect of PA on the viability of Ishikawa cells**


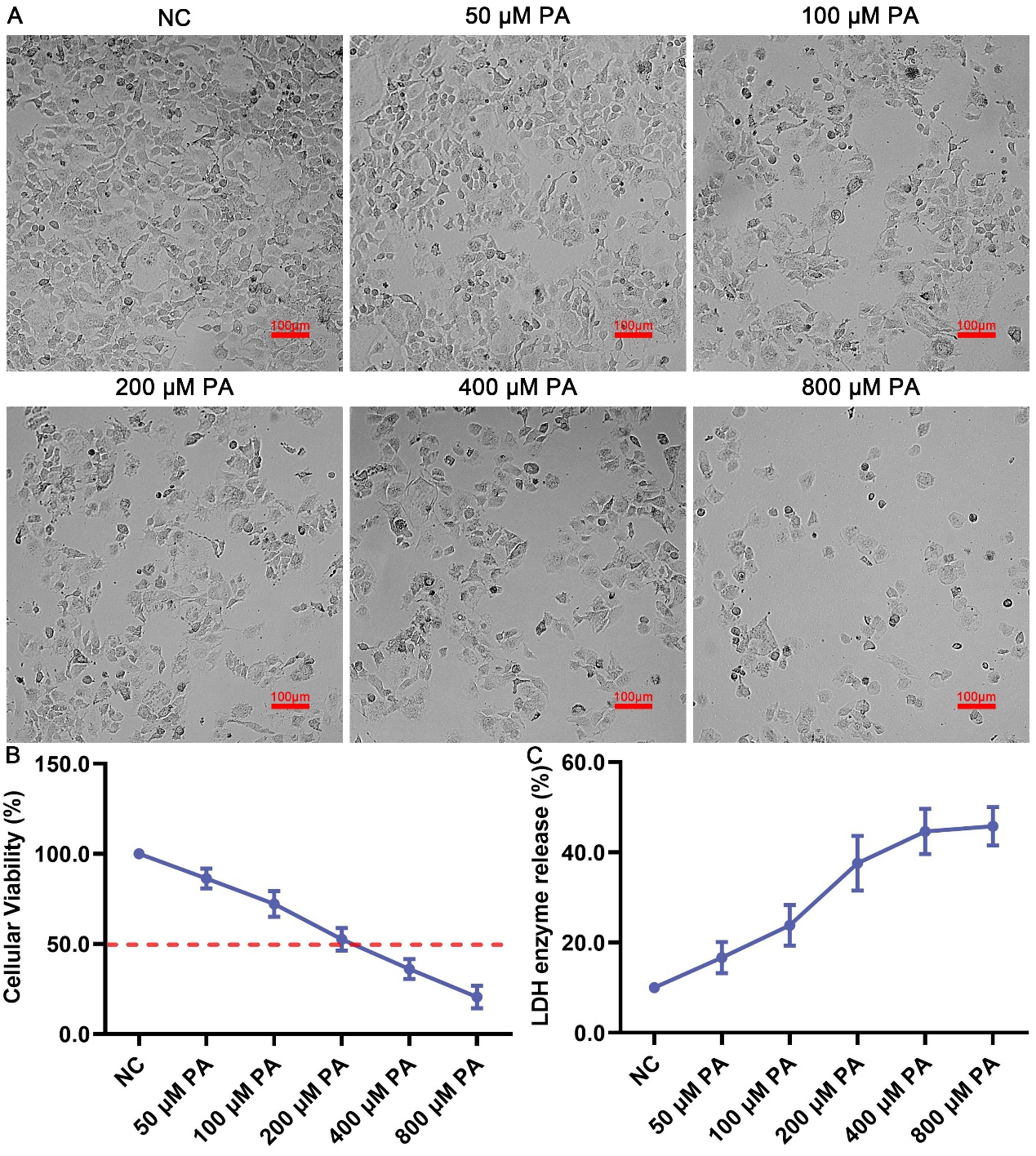


**Supplementation Fig. 2 Effect of Fer-1 intervention on PA-induced inhibition of Ishikawa cell viability**


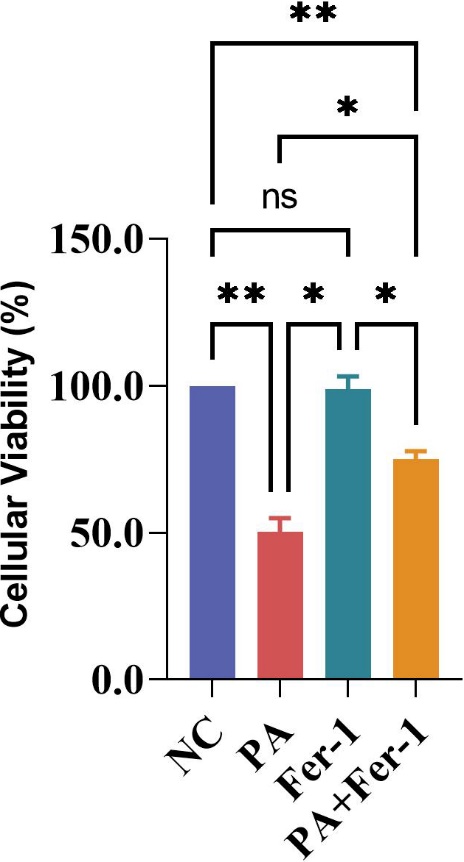


**Supplementation Fig.3 Effect of Fer-1 intervention on the inhibited malignant behaviors of Ishikawa cells induced by PA treatments**


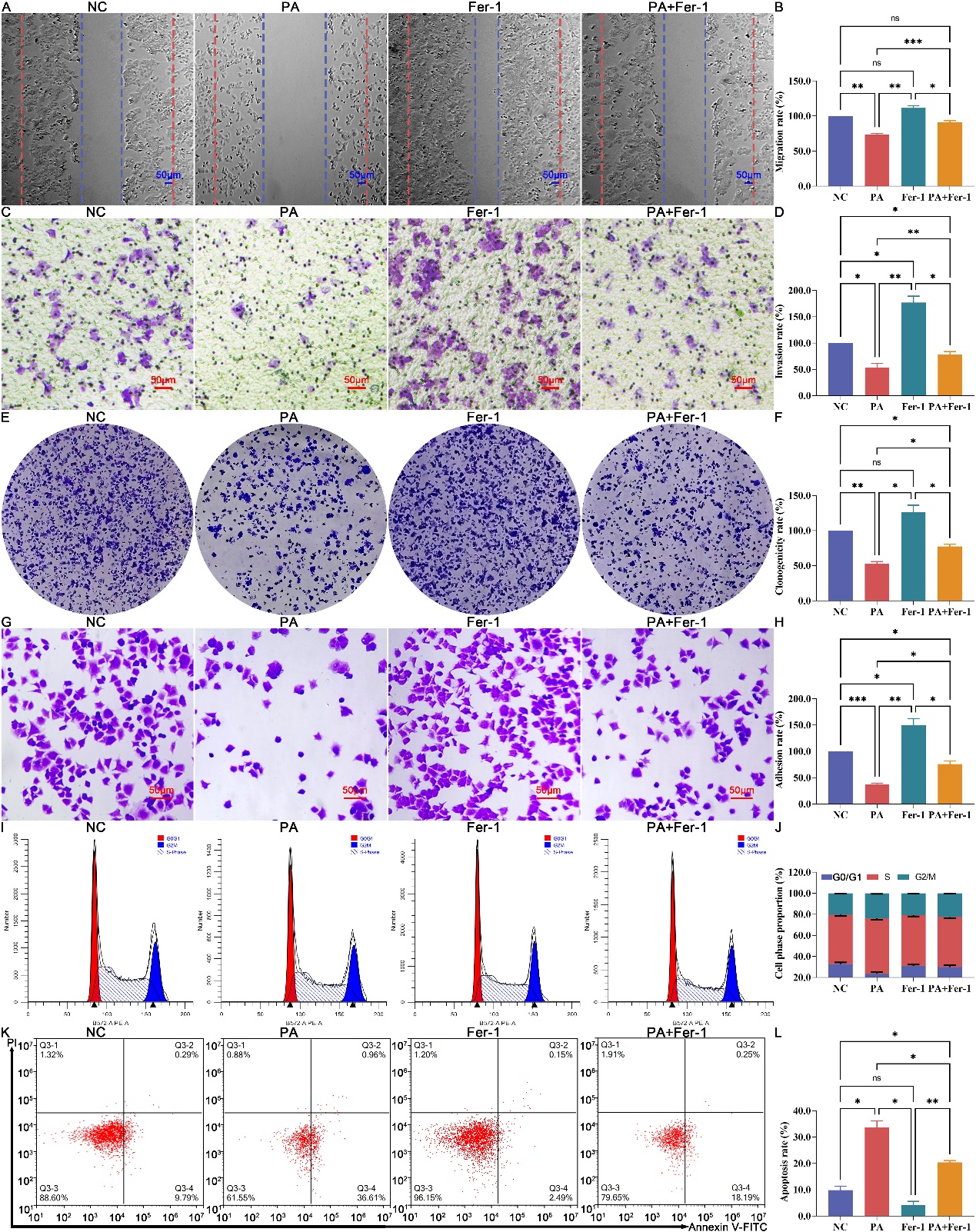


**Supplementation Fig.4 Effect of Fer-1 intervention on the ferroptosis of Ishikawa cells induced by PA treatments**


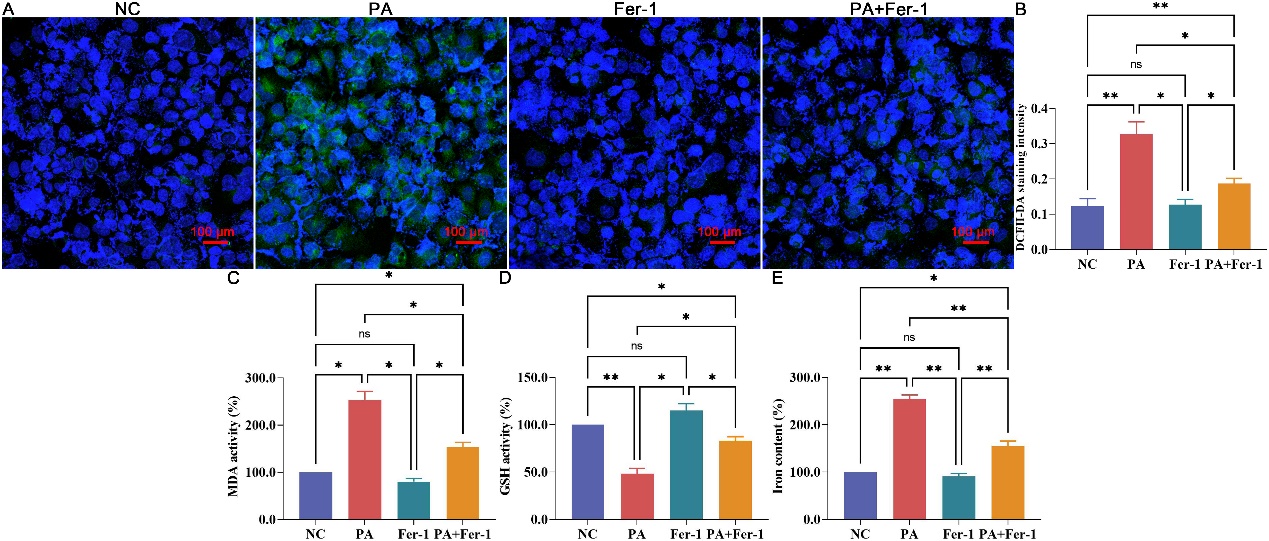

Supplement: Multimedia component 1 [file mmc1.docx]
